# Kidney Biopsy in Type 2 Diabetic Patients: Critical Reflections on Present Indications and Diagnostic Alternatives

**DOI:** 10.3390/ijms22115425

**Published:** 2021-05-21

**Authors:** Domenico Santoro, Massimo Torreggiani, Vincenzo Pellicanò, Valeria Cernaro, Roberta Maria Messina, Elisa Longhitano, Rossella Siligato, Guido Gembillo, Ciro Esposito, Giorgina Barbara Piccoli

**Affiliations:** 1 Unit of Nephrology, Department of Clinical and Experimental Medicine, University of Messina, 98124 Messina, Italy; domenico.santoro@unime.it (D.S.); vincenzo.pellican@gmail.com (V.P.); vcernaro@unime.it (V.C.); robi.messina.rm@gmail.com (R.M.M.); elisa.longhitano@libero.it (E.L.); rossellasiligato@gmail.com (R.S.); guidogembillo@live.it (G.G.); 2 Néphrologie et Dialyse, Centre Hospitalier Le Mans, 194 Avenue Rubillard, 72037 Le Mans, France; gbpiccoli@yahoo.it; 3 Unit of Nephrology and Dialysis, Department of Internal Medicine, ICS Maugeri S.p.A. SB, University of Pavia, 27100 Pavia, Italy; espositociro56@live.it

**Keywords:** diabetic nephropathy, diabetic kidney disease, type 2 diabetes, CKD, renal biopsy

## Abstract

Roughly 3% of patients worldwide with a new diagnosis of type 2 diabetes mellitus (T2DM) already have an overt nephropathy at diagnosis and about 20–30% of the remaining ones develop a complication of this kind later in life. The early identification of kidney disease in diabetic patients is important as it slows its progression, which is important not only because this reduces the need for renal replacement therapy, but also because it decreases the high rate of mortality and morbidity associated with a reduction in kidney function. The increasing prevalence of type 2 diabetes and the consequent greater probability of finding different types of kidney diseases in diabetic patients frequently gives rise to overlapping diagnoses, a definition encompassing the differential diagnosis between diabetic and non-diabetic kidney disease. The issue is made more complex by the acknowledgement of the increasing frequency of presentations of what is termed “diabetic kidney disease” without relevant proteinuria, in particular in T2DM patients. Distinguishing between diabetes related and non-diabetes related forms of kidney disease in diabetic patients is not only a semantic question, as different diseases require different clinical management. However, while the urologic and macrovascular complications of diabetes, as well as overlapping parenchymal damage, can be diagnosed by means of imaging studies, often only a kidney biopsy will make a differential diagnosis possible. In fact, the coexistence of typical diabetic lesions, such as nodular glomerulopathy or glomerulosclerosis, with different glomerular, vascular and tubulo-interstitial alterations has been extensively described, and an analysis of the dominant histological pattern can contribute to determining what therapeutic approach should be adopted. However, due to the high frequency of kidney diseases, and to the fact that T2DM patients are often affected by multiple comorbidities, a kidney biopsy is not generally performed in T2DM patients. What follows is a review aiming to discuss the diagnostic work-up, on the base of clinical, laboratory and imaging criteria, and evaluate the present indications and alternatives to renal biopsy.

## 1. Entity of the Problem

It is well known that type 2 diabetes mellitus (T2DM) and arterial hypertension are the two main causes of chronic kidney disease (CKD) requiring renal replacement therapy.

Globally, the prevalence of diabetes, in particular of type 2 diabetes, has quadrupled in the past three decades, and this disease is now considered the ninth leading cause of death: the estimated prevalence is currently around 10% and is rapidly growing in particular in Asian countries, closely following the obesity epidemic. T2DM accounts for about 90% of total diabetes prevalence [1].

The incidence of diabetic nephropathy in T2DM patients varies widely, depending on genetic background, lifestyle, food habits, socioeconomic status and overall diabetes care. Although genetic predisposition partly determines individual susceptibility to T2DM, obesity, an unhealthy diet and a sedentary lifestyle are important drivers of the current increase in the disease [2].

According to the timing of diagnosis, many if not most T2DM patients present at least one macro- or microvascular complication at diagnosis; most of the remaining patients will develop at least one complication during follow-up. While cardiovascular disease is the main cause of morbidity and mortality in T2DM patients, as well as in the general population of many Western countries, its relative risk is significantly increased in the presence of diabetes [3].

However, less is known about the epidemiology of kidney diseases in diabetic patients, which by itself is associated with an increased risk of mortality and morbidity [4]. Several elements account for this knowledge gap, including differences in defining kidney disease(s) in diabetic patients, differences in screening programs, and competitive mortality.

Furthermore, determining whether a death has been caused by diabetes or kidney diseases is not simple: at least in the Western world, where renal replacement therapy is available, diabetic patients rarely die of “kidney disease”. Within these limits, kidney disease is now considered one of the most important, if not the major cause of mortality and early morbidity in diabetic patients. In 2017 alone, 219,451 deaths globally were attributed to kidney disease in diabetic patients. These deaths accounted for approximately 34% of all deaths of men and 36% of women with kidney diseases. These proportions have increased since 1990, when the corresponding figures were 29% of all CKD-related deaths for men and 32% for women [5].

In a study of 15,046 diabetic patients conducted by the Kidney Research Institute of the University of Washington, CKD was present in 42.3% and 9.4% of individuals with and without type 2 diabetes, respectively. For subjects without diabetes and kidney disease (control group), the standardized cumulative all-cause mortality at 10 years was 7.7%. Standardized mortality was 11.5% for individuals with diabetes but without kidney disease (adjusted for demographics, smoking habits, blood pressure and dyslipidemia) but the combination of diabetes and CKD led to a standardized mortality rate of 31.1%, with an absolute risk difference of 23.4% compared to the control group [4]. 

While the definition of diabetic kidney diseases depends very largely upon the diagnostic parameters used, it is generally appreciated that 20–40% of patients with diabetes develop a clinically relevant nephropathy. These figures can be affected by different definitions (Table 1), but there is general agreement that diabetes is one of the leading causes of CKD and end-stage kidney disease worldwide. Within these limits, in recent decades the prevalence of T2DM has increased almost exponentially in patients who start renal replacement therapy [6,7].

The nomenclature of kidney diseases associated with diabetes has changed remarkably in recent years. Definitions based mainly on progressive increases in proteinuria have been challenged by the increase in clinical forms with no or scant proteinuria associated with typical histological diabetic lesions.

## 2. Frequency of “Non-Diabetic” Kidney Disease in Diabetic Patients

As this review will discuss in greater detail, data in the literature are highly heterogeneous depending on genetic background, the patient’s environment and lifestyle, the organization and quality of diabetes care and, probably most importantly, the different indications for kidney biopsy (Table 2).

Four examples from large studies exemplify this heterogeneity.

In a pivotal study in the United States, Sharma and colleagues evaluated the clinical and histopathological characteristics of patients with type 2 diabetes who had undergone a kidney biopsy between 2011 and 2013 [10]. Of the 2642 biopsies performed, 620 (23.5%) were in diabetic patients. Kidney disease was in stage 4 or 5 in roughly half of the cases. Interestingly, results were almost evenly divided between diabetic nephropathy (37%), non-diabetic kidney disease (36%) and a combination of the above (27%). In patients with isolated, non-diabetic kidney disease, the common diagnoses of proteinuric and acute kidney diseases were represented, as expected, including focal segmental glomerulosclerosis (22%), hypertensive nephrosclerosis (18%) and acute tubular necrosis (17%), followed by IgA nephropathy (11%), and membranous nephropathy (8%). Acute tubular necrosis was more frequent in concomitance with signs of diabetic nephropathy, suggesting that a rapid deterioration in kidney function was a frequent reason for performing a kidney biopsy and that the presence of diabetic lesions may favor acute tubular necrosis. This finding is of potential clinical relevance, since it is well acknowledged that episodes of acute tubular injury are associated with a higher risk of progression to end-stage renal disease, especially in diabetic patients [11,12,13,14].

Another large study analyzing 832 renal biopsies in T2DM patients in Spain reported somewhat different data (non-diabetic nephropathy in about 50% of cases, isolated diabetic nephropathy in 39.5%, and a mixed picture in 10.8%). While all the main proteinuric nephropathies are represented, the low prevalence of acute tubular necrosis (under 5%) is evidence of different biopsy criteria [15].

A recent meta-analysis gathering data on 4876 type 2 diabetic patients with a kidney biopsy reported a very wide range of prevalence of non-diabetic lesions (up to 82.9%) [16]. IgA nephropathy was the most frequent non-diabetic histological diagnosis found in recent studies, in particular those done in Asia, although similar findings have been described in Europe [17,18,19,20].

In a retrospective series from Thailand, Kritmetapak retrospectively analyzed the data of patients with type 2 diabetes who had undergone renal biopsy between 2011 and 2015; it was found that the most frequent indication for renal biopsy was a recent and rapid onset of nephrotic syndrome (41%), followed by a rapidly progressive and unexplained decline in renal function (29%) and the presence of an active urinary sediment (21%) [17]. In this setting, isolated diabetic nephropathy was diagnosed in about half of the cases (51%), while non-diabetic kidney disease (20%) and a combination of the above (29%) accounted for the other half. Besides reporting that the kidney biopsy was prescribed at higher levels of proteinuria in cases later found to have a diabetic nephropathy, the study highlights the importance of urinary sediment analysis, which is reported as more active in patients with non-diabetic or combined kidney diseases.

## 3. From the Clinical Definition of Diabetic Nephropathy to the Present Challenges and Nomenclature

**Figure 1:**
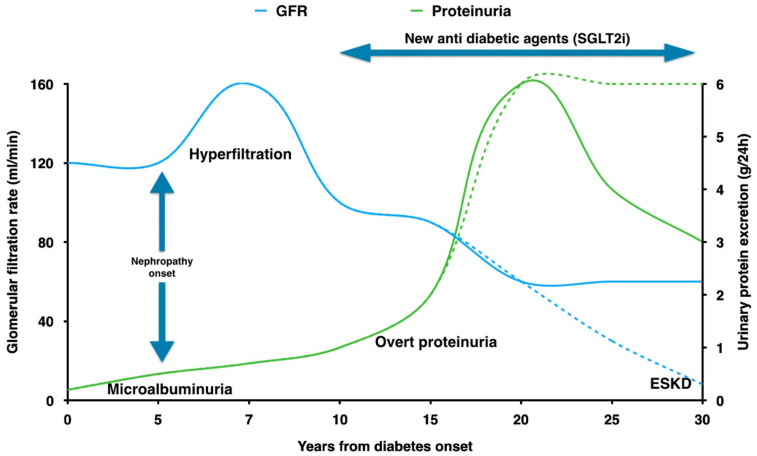
Natural history of diabetic nephropathy, dashed lines (modified from [8]): after an initial phase of hyperfiltration at the onset of diabetic kidney disease, the kidney function (glomerular filtration rate) slowly decreases over the years as proteinuria increases. This hyperfiltration phase is often missed and patients are referred to nephrologists once the glomerular filtration rate is already reduced or an overt proteinuria develops. Solid lines represent the modulation of the natural course of diabetic kidney disease by new treatment options. GFR: glomerular filtration rate; SGLT2i: sodium glucose transporter 2 inhibitors; ESKD: end-stage kidney disease.

The classic diagnosis of “diabetic nephropathy” was based on the presence of persistent proteinuria, slowly evolving from a stage of microalbuminuria. “Macro” albuminuria classically preceded the development of hypertension, and was followed by a progressive decline in kidney function (Figure 1) [8].

The duration of diabetes was a critical issue, and it was generally held that the interval before the development of the first signs of diabetic nephropathy was about 10 years in general, an interval that could be modulated by the level of glycemic control (the better the control, the longer the interval). An ancillary but very important diagnostic criterion was the presence of diabetic retinopathy (“diabetic renal retinal syndrome”) [21,22,23].

It is interesting to note that this clinical sequence was described in a context of a standard treatment of diabetes which encompassed twice daily rapid-acting insulin administrations, before the diabetes control and complications trials (DCCT) study shifted “basic” diabetes control to three injections a day [24]. Therefore, it is not surprising that important changes in diabetes care, from long-acting insulin to its use in combination with oral drugs, also changed the natural history of the disease in all diabetic patients. 

The Mogensen sequence is still an important reference in type 1 diabetes patients, in particular if their metabolic control has been poor for long periods. Even in these cases, however, the natural history of the disease has been modulated by new methods of treatment, with an overall longer interval from diagnosis to overt CKD, and a lower incidence of patients with a full-blown nephrotic syndrome. Long-term optimal glycemic control is still the best way to reduce the risk of developing CKD and slowing its progression to end-stage kidney disease [25]. However, what is true at the population level may not necessarily be true at the individual patient level, and clinical dissociations are not rare. Newly emerging phenotypes of diabetic kidney disease have recently been described: non-albuminuric renal impairment and progressive renal decline [26,27,28]. 

**Figure 2:**
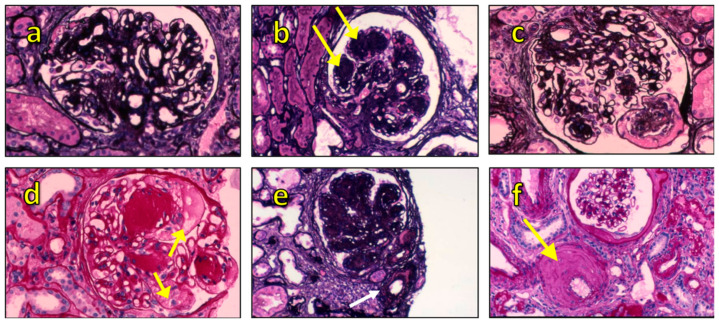
Morphological spectrum of diabetic nephropathy: (**a**) diffuse mesangial sclerosis. Periodic Schiff-Methenamine (PASM) staining 400×; (**b**) nodular glomerulosclerosis (arrows), also called Kimmelstiel–Wilson nodules. PASM staining 400×; (**c**) disintegration of mesangial matrix which presents fibrillar aspects. First step of nodule formation. PASM staining 400×; (**d**) glomerular microaneurysms (arrows) may arise in association with mesangiolysis due to a loss of anchoring points between GBM and the mesangium. PASM staining 400×; (**e**) afferent and efferent glomerular hyalinosis at vascular pole (arrow). Often present in diabetes, but not specific. PASM staining 200×; (**f**) Intimal fibrosis with reduplication of elastic lamina (arrow). PASM staining 200×. Images by D.S.

The discrepancy between clinical presentation and morphologic damage may be impressive, and morphologic lesions typical of diabetic nephropathy (Figure 2) can be found even in the presence of normal or slightly reduced kidney function without proteinuria [26,27,28,29]. This particular phenotype of diabetic kidney disease without relevant proteinuria was already described almost 30 years ago [30,31]. However, a large body of recent evidence, suggests that, nowadays, 8–16% of type 2 diabetic kidney disease patients present without relevant proteinuria [26].

These considerations apply to an even greater degree to patients with type 2 diabetes, including cases in which the natural history is often less clear, the diagnosis is frequently concomitant with the discovery of end-organ damage and it is difficult, and sometimes impossible, to distinguish between diabetes as a comorbidity and diabetes as a cause of kidney disease [28,32].

Diabetes and obesity frequently go together [33]; diabetes, hypertension and vascular diseases are likewise so closely associated that distinguishing the relative role of each element is almost impossible. The emerging entity of non-proteinuric diabetic nephropathy, in which advanced glomerular lesions may coexist with scant or no proteinuria, has challenged our previous interpretations [34,35]. 

The well-known limits in the clinical definition of diabetic nephropathy in type 2 (vs. type 1) diabetes are summarized in Table 3.

## 4. Diabetic Nephropathy, Nephropathy in a Diabetic Patient or Diabetic Kidney Disease?

In particular in type 2 diabetes, due to the frequent coexistence of diabetes, obesity and hypertension, within the panoply of the metabolic syndrome, some authors feel that the term “diabetic nephropathy” should be replaced by “kidney diseases in diabetic patients”, as the latter highlights the variety of potential lesions and the importance of diabetes as a comorbid condition in conjunction with other kidney diseases.

The emerging term “diabetic kidney disease” highlights the protean nature of the kidneys’ involvement in diabetic patients: tubulo-interstitial and vascular lesions coexist with a variegated picture in which the classical nodular Kimmestiel–Wilson lesions are progressively losing their role in favor of less specific glomerlosclerotic lesions [36]. Conversely, although rare, the same lesions are described in non-diabetic patients, outside the context of myeloma and other recognized causes of nodular (albeit differentially stained) lesions.

The definition of the focal and segmental lesions of diabetic glomerulosclerosis is likewise not fully clear, and since focal segmental glomerulosclerosis is often both cited as a diabetic lesion and listed as a non-diabetic lesion in diabetic patients, this may be adding to the discrepancies in the definition of what is diabetic and what is non-diabetic kidney disease [37].

While the same glomerular lesions are occasionally found in non-proteinuric diabetic patients, a pragmatic distinction between proteinuric and non-proteinuric kidney diseases in diabetic patients has been suggested by some authors, as an alternative to the attempted distinction between diabetes as a cause of kidney disease and diabetes as a comorbidity [38,39,40]. In fact, in two recent large cohorts of elderly patients with advanced kidney disease being followed in Italy and France the difference in outcomes was more linked to overall comorbidity and the presence of proteinuria than to diabetes per se [38,41]. Furthermore, a large analysis of about 2000 patients on follow-up in a large French center highlighted how the combination of clinical diagnoses of diabetic kidney disease and nephroangiosclerosis dominates the picture in elderly patients [42]. Once more, the prevalence of non-diabetic versus diabetic kidney disease is determined both by how frequently these patients (usually ones affected by non-proteinuric diseases) undergo a kidney biopsy and by the consideration that different types of vascular lesions may be associated with diabetes. In this regard, the issue is closely linked to the indications for a kidney biopsy in the diseases clinically defined as nephroangiosclerosis, which have recently been undergoing critical reevaluation [43,44].

## 5. Why Is a Kidney Biopsy Needed (or Is a Biopsy Not Always Needed)?

While a kidney biopsy remains the gold standard not only for diagnosis but also for defining prognosis in many types of glomerular diseases, it is always an invasive procedure, in particular in high-comorbidity patients, which diabetic patients often are [45,46]. It is noteworthy that in a recently published large French survey encompassing 52,138 patients who had a percutaneous native kidney biopsy between 2010 and 2018, the presence of diabetes and obesity were not associated with a higher risk of relevant bleeding (requiring at least one blood transfusion, and reported in 5% of the cases), and had, almost paradoxically, a protective score [27].

The classic tenet is that a kidney biopsy is indicated in cases when there is a need for a differential diagnosis that might change the clinical management of a diabetic patient. The current indications are still affected by therapeutic minimalism, at least in part a heritage from the past decades, when the treatment of glomerulonephritides was generally steroid-based, and the presence of diabetes was considered a contraindication to such treatment. Advances in glomerular disease care and the extraordinary development of alternative non-steroid based schedules challenge this minimalism and may favor the kidney biopsy.

At present the main indications can be summarized as follows: the differential diagnosis of a glomerulonephritis—glomerular involvement (primitive or in the context of a systemic disease) and the presence of rapidly progressive or sudden impairment in kidney function. As previously discussed, the relative frequency of non-diabetic kidney diseases found in the kidney biopsy correlates to a high degree with the way these classic indications are interpreted (Table 2). As a consequence, the wider the indications, the higher is the prevalence of typical diabetic nephropathy cases. Since only a small minority of diabetic patients undergo a kidney biopsy, the discussion about whether the different frequencies of non-diabetic nephropathy reflect their prevalence in the general population (for example IgA nephropathy in Asia and in the Mediterranean countries), different indications for kidney biopsy, or different diabetes care, is destined to remain open.

These classic tenets are linked to a glomerular-centric, glomerulonephritis-centric vision, and may be challenged as more is learned about the development and course of non-proteinuric (or not intensely proteinuric) diabetic nephropathy. Likewise, recent therapeutic advances are likely to contribute to guiding clinical management [47,48].

## 6. Effect of the Indications for a Kidney Biopsy and Potential Diagnostic Alternatives

When the kidney biopsy is driven by the presence of heavy proteinuria or its rapid onset, a high prevalence of membranous nephropathy, focal segmental glomerulosclerosis and secondary nephropathies linked to myeloma-amyloidosis are expected, in particular in elderly patients. Conversely, if attention is focused on unexplained hematuria, another classical indication for a kidney biopsy, IgA nephropathy will dominate the picture. 

Acute tubular necrosis, interstitial nephropathies and, less frequently, rapidly progressive glomerular diseases, will be involved whenever the indications are linked to an unexplained rapid fall in glomerular filtration rate. The first two forms dominate in cases in which the kidney biopsy is suggested and in which there is scant or tubular-only proteinuria (Table 2).

Even in these cases, the advances being made in diagnosis and definition of kidney diseases may lead to diagnosis without the need for a kidney biopsy, and thus modulate the prevalence of specific diseases. Due to the widespread clinical use of phospholipase A2 receptor (PLA2R) antibodies, primary membranous nephropathy is a diagnosis that does not necessarily demand a kidney biopsy, relying on histological definition more for prognostic indications than for diagnostic ones [49,50]. 

The case of focal segmental glomerulonephritis is complex: this nosological entity is interpreted more as a common lesion than as a separate disease, and, in this context, the cases connected with a disproportion between kidney mass and total body mass (reduction in nephron mass, obesity, etc.) are increasingly frequent [51]. A diagnosis can be reached only if a kidney biopsy is carried out. In cases with negative PLA2R antibodies, without evidence of monoclonal gammopathy or full blown nephrotic syndrome, and in the presence of obesity and a reduction in kidney mass, the odds that a kidney biopsy will find focal and segmental glomerulosclerosis are high, and the chances that the diseases is amenable to specific care are probably limited. 

Likewise, the presence of tubular proteinuria, the temporal relationship with new drugs or the use of drugs known to be associated with chronic interstitial nephropathy in the long run may be the clues that make it possible to diagnose acute and chronic tubulointerstitial diseases. 

**Figure 3:**
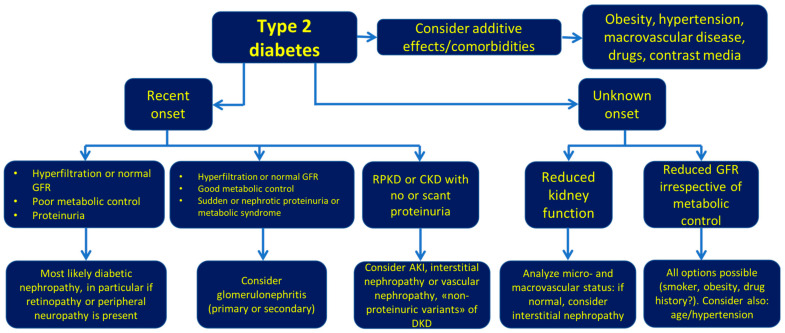
Diagnostic hypotheses flow-chart of diabetic kidney disease modified from [28]. Consider kidney biopsy in case of nephrotic proteinuria without retinopathy and in non-obese patients with sudden onset of proteinuria or stepwise increase of serum creatinine, in particular if the vascular status is well preserved and with no or scant proteinuria. RPKD: rapidly progressive kidney disease.

In the absence of specific biomarkers, the acknowledgement that a family history of IgA nephropathy is present in about 30% of cases is an additional diagnostic clue in cases in which the main reason for a kidney biopsy is micro hematuria. In this context, the interest in performing a kidney biopsy for patients with a family history of IgA nephropathy or those with Henoch–Schönlein disease, is probably limited to cases with an active urinary sediment or a relevant proteinuria, in which a therapeutic option is being considered. Once more, the context is extremely sensitive, and the present widespread minimalism towards immunologic treatments in IgA nephropathy tends to further limit the indications in the cases without a rapid reduction in kidney function (Figure 3).

The importance of dysmorphic micro hematuria has been highlighted in some studies [52].

The presence of dysmorphic red blood cells (over 80% of the erythrocytes) was found to be significantly correlated with a diagnosis of non-diabetic kidney disease, in a series from China encompassing 198 kidney biopsies in T2DM patients. The difference was statistically significant, and the presence of glomerular hematuria demonstrated high specificity and positive predictive value (0.97 and 0.94, respectively). These data were confirmed by a meta-analysis performed by Jiang et al. [53].

A recent retrospective multicenter study in 832 Spanish T2DM patients found that older age, microhematuria and absence of diabetic retinopathy were independently associated with the risk of having a non-diabetic kidney disease [15]. Moreover, T2DM patients with diabetic kidney disease or mixed forms had a worse prognosis and higher chances of disease progression to ESKD.

While discussing the role of renal imaging is beyond the scope of this review, it should be noted that it can lend support to an alternative or additive diagnosis, in particular in cases of important reduction in the nephron mass, such as in the context of a functionally single kidney, of congenital, vascular, or urologic origin. Renal imaging also makes it possible to evaluate the potential risks involved in a kidney biopsy: besides the obvious considerations in the case of a single kidney or in the presence of renal cysts, the risk of bleeding tends to be higher in the presence of small kidneys with high resistive indexes, and the potential benefit of a specific therapy may be lower in such cases. 

Non-renal imaging is also of interest; besides the importance of the definition of cardiovascular morbidity, including cardiac imaging (ultrasounds, nuclear magnetic resonance and analysis of the calcium score at cardiac level, as well as of calcifications of the peripheral and renal artery) the evaluation of easily accessible districts, such as the carotids, can help us target questions and contribute to diagnosing nephroangiosclerosis and atherosclerosis related diseases [54].

A vast array of urinary markers has been tested in diabetic patients in the last decade. In addition to albumin, these include cystatin-C, angiotensinogen, kidney injury molecule 1 (KIM-1) and neutrophil gelatinase-associated lipocalin (NGAL). The soluble form of the urokinase plasminogen activator receptor (suPAR) seems to be particularly promising and it has recently been proposed as an early marker of future kidney disease in diabetic patients [55]. In fact, suPAR levels in diabetic patients are not only elevated, but seem to predict microalbuminuria several years before its onset [56,57]. It is likely that these markers will contribute more to improving prognostic rather than diagnostic definition [58,59,60,61]. Likewise, the promises of metabolomics have not yet been fulfilled, but the definition of a “metabolomic signature” of the different nephropathies in diabetic patients opens interesting future perspectives [62,63,64,65]. Unfortunately, despite the number of candidate biomarkers available, their use in routine clinical practice is still limited [66,67]. 

Overall, since so far most diagnostic clues have been seen to be in the patient’s clinical history, and in careful clinical phenotyping of the patient, wide use of the above considerations and integrated tools, from history to imaging and urinalysis, could once more affect the relative prevalence of diabetic and non-diabetic kidney disease in the hands of skilled physicians, and in settings in which strict chronic care of diabetic patients is the rule. 

## 7. Alternative and Emerging Indications for a Kidney Biopsy in Diabetic Patients

There are three further, emerging or reasonably well-established indications for a kidney biopsy in diabetic patients, partially shared by non-diabetic patients with non-intensely-proteinuric kidney disease: (1) the identification of prognostic markers; (2) the diagnosis of typical diabetic nephropathy without proteinuria; (3) the precise identification of the patient’s kidney disease in view of kidney transplantation, or with regard to the use of novel diabetes therapies that are potentially effective in improving kidney function [35,68,69].

In a recent review on renal structure in diabetic patients, Fioretto and coworkers describe the many advantages of a histological diagnosis in T2DM patients: (1) better definition of the renal prognosis, (2) a better understanding of the different clinical phenotypes and of different responses to therapies, and, when possible, (3) being able to reassure patients with minimal diabetic nephropathy lesions that their risk of substantial loss of GFR in the near future is low [70]. Due to the complex balance between reassurance and fear of the kidney biopsy, as well as the desire to avoid unnecessary hospitalizations, this patient-centered goal is likely to be highly context sensitive.

While kidney biopsy, in particular in the presence of a small sample size, is not a precise marker of prognosis, it is well known that the presence and extent of the lesions it reveals correlate with prognosis; this is true in particular in the case of interstitial fibrosis, which is a common element in diabetic kidneys, and which is closely associated with disease progression. Similar considerations generally apply to vascular damage, although its assessment is less standardized, and to glomerular involvement [71,72,73].

Within these limits, a clinical reading of the kidney biopsy can support clinical reasoning. For instance, the finding of predominantly tubulo-interstitial damage draws attention to the usually heavy polypharmacy used to treat diabetic patients [74,75]. The list of potential culprits is long, but since some of them, including antibiotics, acetaminophen, allopurinol, H_2_ receptor antagonists, proton pump inhibitors, loop diuretics and thiazides are widely used in diabetes, the acknowledgement of their potential role could lead to reconsidering the cost–benefit ratio of multiple therapy versus attempting to correct most, if not all metabolic alterations, especially in advanced disease [76].

On the other hand, the presence of significant vascular involvement could lead to a modulation of antihypertensive drugs and eventually of statins. The presence of florid glomerular involvement suggests the importance of reducing hyperfiltration stress, combining blood pressure and glycemic control with weight reduction, modulation of protein intake and the prompt introduction of the new hypoglycemic agents with a favorable kidney profile. Recently, sodium-glucose transporter 2 inhibitors (SGLT2i) have been shown to exert powerful nephroprotective effects through a variety of mechanisms [77]. The early trials designed to assess the efficacy of these drugs on glycemic control demonstrated a reduction in proteinuria and renal disease progression [78,79,80,81]. Later studies confirmed these associations and opened the way for the use of SGLT2i even in non-diabetic kidney disease [82,83,84]. These trials represent the most important breakthrough for the treatment of diabetic nephropathy since the introduction of ACE inhibitors and could potentially modify the natural history of the disease as we presently know it (Figure 1).

## 8. Mathematical Approaches and Risk Scores

Efforts to combine the considerations mentioned above into risk scores able to contribute to selecting patients with non-diabetic nephropathy for a kidney biopsy led to the proposal of mathematical models [17,54,85,86,87]. All these scores are based on studies that sought to weigh the effect of well-known and partly interdependent factors associated with DKD: duration of diabetes, systolic blood pressure, glycated hemoglobin, presence of hematuria and diabetic retinopathy, and, in some cases, the entity of proteinuria [17,87].

As previously discussed, interest in these models is probably at least partially offset by the deep revision of the therapeutic approaches to the care of diabetes and its complications, which may increase interest in clarifying the definition of the forms of kidney involvement that were previously considered not amenable to any specific therapy [47,48,88].

## 9. Take Home Messages

According to WHO data, published in 2012, about 347 million people had a diagnosis of diabetes mellitus, which today represents the main risk factor for CKD and ESRD in the Western world [89,90]. About 30–40% of diabetic patients develop CKD after 10–15 years of diabetes. However, diabetic nephropathy is not the sole disease, and up to 50% of these patients may have a different kidney disease and may benefit of targeted therapy. When balancing the risk to benefit ratio of an histological assessment, consider performing a renal biopsy in case of discrepancy between kidney and other end-organ damage, rapidly progressive kidney function impairment, sudden development of nephrotic proteinuria, in particular in the presence of a good metabolic control. 

## Figures and Tables

**Table 1:** Definitions of diabetic and non-diabetic nephropathy in diabetic patients.

Definition	Comments
Diabetic nephropathy (DN)	Classic definition of a chronic kidney disease with a progressive increase in proteinuria and hypertension, up to end-stage kidney disease. Microvascular lesions coexist [8].
Diabetic kidney disease (DKD)	Presently the preferred definition of diabetic nephropathy. Although more widely used, it is commonly used as a synonym of DN or chronic kidney disease attributable to diabetes mellitus (as in this paper) [9].
Nephropathy in a diabetic patient	General term encompassing all types of CKD in a diabetic patient, including nephroangiosclerosis, obstructive nephropathy, etc.

**Table 2:** Kidney biopsy indications in a diabetic patient and most likely histological findings other than diabetic nephropathy.

Main Indication for Kidney Biopsy	Major Expected Histological Findings
Isolated microscopic hematuria	IgA nephropathy, especially in young patients of Asian and Mediterranean origin
Rapid onset of nephrotic syndrome	Membranous nephropathy, especially in elderly patients Monoclonal gammopathy-related nephropathies
Progressive onset of nephrotic syndrome	Obesity related glomerulopathy (may be difficult to distinguish from DN) Focal-segmental glomerulosclerosis in its secondary forms Nephroangiosclerosis/vascular involvement
Rapidly progressive or stepwise reduction in kidney function with relevant proteinuria	Rapidly progressive glomerulonephritis Atheroembolic disease
Rapidly progressive or stepwise reduction in kidney function with scant proteinuria	All forms of acute kidney injury Vascular nephropathy Interstitial nephropathy
Non-proteinuric chronic kidney disease	Classic nephroangiosclerosis, in particular in hypertensive patients or in patients with a history of heavy smoking

**Table 3:** Clinical differences between type 1 and type 2 diabetic patients presenting a diabetic kidney disease.

Clinical Criteria	Type 1 Diabetic Patients	Type 2 Diabetic Patients
Duration of diabetes mellitus	Clearly identified in most cases	Unclear in most patients due to the frequent lag between onset and diagnosis
Glycemic control	Known since the start of specific therapy (insulin)	Unknown in the period preceding the diagnosis
Onset of hypertension	Usually after diagnosis	Frequently preceding diagnosis
Retinopathy	Usually present at CKD diagnosis	Cataract may be more prevalent
Microscopic hematuria	Rare, non-typical	Frequent (hypertension, other causes in elderly patients)
Peripheral neuropathy	Usually identifiable at CKD diagnosis in its typical form	Carpal tunnel syndrome may be more typical

## Data Availability

No new data were created or analyzed in this study. Data sharing is not applicable to this article.
